# Antifungal Resistance: Specific Focus on Multidrug Resistance in *Candida auris* and Secondary Azole Resistance in *Aspergillus fumigatus*

**DOI:** 10.3390/jof4040129

**Published:** 2018-12-05

**Authors:** Sevtap Arikan-Akdagli, Mahmoud Ghannoum, Jacques F. Meis

**Affiliations:** 1 Department of Medical Microbiology, Mycology Laboratory, Hacettepe University Medical School, TR-06100 Ankara, Turkey; 2 Center for Medical Mycology, Department of Dermatology, University Hospitals Cleveland Medical Center, Case Western Reserve University, Cleveland, OH 44106, USA; mag3@case.edu; 3 Department of Medical Microbiology and Infectious Diseases, Canisius Wilhelmina Hospital (CWZ), 6532 Nijmegen, The Netherlands; jacques.meis@gmail.com; 4 Centre of Expertise in Mycology Radboudumc/CWZ, 6532 Nijmegen, The Netherlands

**Keywords:** *Candida auris*, *Aspergillus fumigatus*, antifungal resistance, multidrug resistance, mechanisms of antifungal resistance

## Abstract

Antifungal resistance is a topic of concern, particularly for specific fungal species and drugs. Among these are the multidrug-resistant *Candida auris* and azole-resistant *Aspergillus fumigatus*. While the knowledge on molecular mechanisms of resistance is now accumulating, further data are also available for the clinical implications and the extent of correlation of in vitro resistance to clinical outcomes. This review article summarizes the epidemiology of *C. auris* infections, animal models focusing on the activity of novel antifungal compounds in *C. auris* infections, virulence factors, and the mechanisms of antifungal resistance for this multi-resistant *Candida* species. Regarding *A. fumigatus*, the significance of azoles in the treatment of *A. fumigatus* infections, reference methods available for the detection of resistance in vitro, molecular mechanisms of secondary azole resistance, routes of acquisition, and clinical implications of in vitro resistance are covered to provide guidance for the current status of azole resistance in *A. fumigatus*.

## 1. *Candida auris*

### 1.1. Epidemiology and Risk Factors for Candida auris Infection

Nosocomial infections with resistant *Candida* species are increasing and candidemia is becoming a public health concern in Europe, the Americas, and Asia. This is associated with increasing numbers of immunocompromised individuals, the rampant empirical use of broad-spectrum antibiotics and fluconazole, and the widespread use of implanted medical devices. Invasive non-*albicans* candidiasis was mainly reported, until recently, due to *C. glabrata*, *C. parapsilosis*, *C. tropicalis*, and *Pichia kudriavzevii* (*C. krusei*). *C. parapsilosis* is common among newborns, while *C. glabrata* is more prevalent among older adults and patients with cancer. *C. tropicalis*, on the other hand, is more commonly seen in patients with leukemia and neutropenia. *C. parapsilosis*, a skin colonizer, is a common pathogen in intravascular catheter-related infections. *Pichia kudriavzevii*, in turn, is found more often among patients with leukemia and associated neutropenia, who receive fluconazole prophylaxis [1]. A new species, *C. auris*, associated with resistance to several antifungal drugs and difficulty in identification, has been observed to be emerging in the last decade. This yeast was first described in East Asia in 2009, after being isolated from a Japanese patient with otitis externa and three Korean patients with candidemia [2,3]. These observations did not attract much attention from the medical community at the time until clonal outbreaks were observed in several Indian hospitals [4,5]. Shortly after these seminal publications, reports followed from Kuwait, South Africa, and Venezuela [6,7,8,9,10]. *C. auris*, which was never heard of prior to the first publication in 2009, became an emerging global health threat with outbreaks occurring in many health facilities. It is highly likely that *C. auris* was an underreported infection in the first years after 2009 due to difficulties in identification [11,12,13,14,15,16]. At present, infected and colonized patients have been identified in Australia [17], Austria [18], Belgium [19], Canada [20], China [21,22,23], Colombia [24,25], Egypt (unpublished), France [19], Germany [19], India [4,26,27,28,29], Iran (unpublished), Israel [30,31], Kenya [15], Kuwait [7,9,10], Korea [3,32], Malaysia [33], the Netherlands [15], Norway [19], Oman [34,35], Pakistan [36], Panama [37], Russia [15], Saudi Arabia [38], Singapore [39], South Africa [8,40], Spain [41], Switzerland [42], Thailand [15], United Kingdom [43,44,45,46], United States [36,47], United Arab Emirates [48], and Venezuela [6]. More than 4000 cases of infection and colonization, the majority from India and South Africa, have been recorded to date, but it is highly likely that we are observing only the tip of the iceberg. 

*C. auris* is a novel *Candida* species in the *Candida haemulonii* species complex, which causes a wide range of infections, especially in debilitated patients residing in intensive care units (ICUs). A large 18-month prospective study in Indian ICUs recorded 1400 candidemia cases; *C. auris* was identified as the fifth most common cause found in 19 out of 27 ICUs, with a prevalence of 5.3% [27]. In some tertiary care Indian hospitals, *C. auris* is the second most common cause of candidemia after *C. tropicalis* [49]. A tertiary medical center in South America reported *C. auris* as the sixth most common cause of nosocomial bloodstream infections between March 2012 and July 2013 [6]. The mode of spread within the hospital setting is through person to person transmission and via contaminated surfaces and/or equipment. During outbreaks, *C. auris* can contaminate the room of colonized or infected patients [50]. It is therefore of utmost importance to quickly identify contaminated surfaces and screen specimens of patients. Real-time detection and identification of *C. auris* is the target of several molecular kits [51,52,53,54,55,56,57,58,59]. The survival of *C. auris* for weeks, even months, within the hospital confirms the importance of infection prevention programs [60,61,62]. Transmission from patient to patient has been documented to lead to skin colonization by *C. auris* and increased risk for candidemia. The hospital environment represents a reservoir that contributes to the nosocomial transmission of *C. auris* similar to that seen with multi-resistant bacterial pathogens [63,64]. Risk factors for infection with *C. auris* are related to immunosuppression, hospitalization in intensive care units over prolonged periods, use of central venous and urinary catheters, and empirical use of antibiotics or antifungals. Adults are mainly affected, but in an outbreak situation in Venezuela 13/18 cases were pediatric patients [6]. As observed in many other studies, all isolates were initially mis-identified as *Candida haemulonii*, a commonly reported mistake [65,66,67]. Sequencing of the internal transcribed spacer (ITS) region and MALDI-TOF analysis were necessary to identify isolates of the outbreak involved as *C. auris*. The predisposing risk factors for *C. auris* infection are similar to other opportunistic *Candida* species [1]; that is, immunocompromised patients (diabetes mellitus, malignancy, chronic renal disease, neutropenia, HIV), concomitant bacteremia, broad spectrum antibacterial or antifungal therapy within 90 days, surgery within 90 days, presence of central venous catheters or urinary catheters, ICU stay, and parenteral nutrition (PN) administration confer an increased risk of acquiring *C. auris*. A case-control study in an Indian center was conducted to determine specific risk factors predisposing to *C. auris* candidemia [29]. Patients with *C. auris* (*n* = 74) and non-*auris* (*n* = 1087) fungemia cases were analyzed. Multivariate analysis showed that patients with respiratory diseases, vascular surgery, and prolonged exposure to fluconazole were more likely to develop ICU-onset *C. auris* fungemia. In describing the epidemiology of *C. auris* infections, the Center for Disease Control (CDC) used whole genome sequencing of 54 isolates collected from India, Pakistan, South Africa, Japan, and Venezuela [36]. Four distinct geographical clades were observed, suggesting emergence at the same time on three continents. Similar geographic clustering was observed with Amplified Fragment Length Polymorphism(AFLP) and proteomic analysis of *C. auris* isolates from three different continents—Asia, Africa, and Latin America [68]. A recent Whole Genome Sequencing (WGS) and single-nucleotide polymorphism (SNP) analysis of *C. auris* strains isolated in the USA showed multiple introductions of *C. auris* isolates belonging to the four clades, and spread among healthcare facilities [47]. Most *C. auris* strains (>60–90%) are resistant to fluconazole, 10–30% exhibit a high minimum inhibitory concentration (MIC) for amphotericin B, and <5% can be considered resistant to echinocandins [28,69]. Given the recent unprecedented worldwide spread and multidrug resistance, *C. auris* is included in the world’s 10 most feared fungi [70].

### 1.2. Virulence Factors of C. auris

To determine the virulence properties of *C. auris* relative to *C. albicans*, a set of clinical strains were investigated regarding the ability to germinate, adhere, and produce extracellular enzymes [71]. *C. auris* strains failed to germinate but, in contrast and as expected, *C. albicans* germinated profusely. Similarly, *C. auris* exhibited a significantly reduced ability to adhere to silicon elastomer disks relative to *C. albicans*. Moreover, the *C. auris* isolates produced phospholipase and proteinase in a strain-dependent manner (37.5% of the *C. auris* strains possessed phospholipase activity, while 64% evaluated secreted proteinase activity). The last virulence factor evaluated was the ability of *C. auris* to form biofilms. Our data showed that the formed biofilms were mainly composed of yeast cells, while biofilms formed by *C. albicans* had a heterogeneous architecture of biofilms comprised of yeast and hyphae morphology embedded within the extracellular matrix. Furthermore, *C. auris* biofilms had a limited amount of extracellular matrix relative to *C. albicans* and its biofilm thickness was significantly less than the biofilms formed by *C. albicans*. Taken together, these data show that *C. auris* is relatively less pathogenic than *C. albicans*.

### 1.3. C. auris Animal Models and Activity of Experimental Antifungals

To gain insight into the in vivo virulence of *C. auris*, an immunosuppressed murine model was developed [72,73]. Once the model was established it was used to evaluate the efficacy of two experimental antifungals (rezafungin and APX001A). The data showed that rezafungin had a significantly reduced CFUs/g kidneys fungal burden compared with vehicle- or amphotericin B-treated groups. Furthermore, treatment with rezafungin resulted in a significantly lower CFUs/g tissue fungal burden compared to micafungin-treated animals [72].

Evaluation of the efficacy of APX001 using the optimized immunocompromised mouse model showed that treatment with this experimental drug resulted in a significant increase in animal survival (between 80 and 100% survival in the three treatment groups). In contrast, treatment with anidulafungin led to only a 50% survival rate. In addition, APX001 treatment led to a significant reduction in CFUs/g of kidneys, lung, and brain tissue compared to the vehicle-treated group [73]. In an immunocompetent murine model, virulence was also highest for *C. albicans*, closely followed by *C. auris*, *C. glabrata*, and *C. haemulonii*, respectively [74].

### 1.4. Resistance of C. auris

Besides being antifungal-resistant, *C. auris* is thermotolerant, grows well up to 42 °C, and is salt-tolerant (up to 10%). These characteristics can be used to design selective media for the detection of *C. auris* for screening purposes which have been used successfully in outbreak investigations [75]. Concerning resistance to antifungal agents, *C. auris* has demonstrated extensive resistance to azoles and amphotericin B [24,28]. The ATP-binding cassette (ABC) transporter activity was significantly higher in *C. auris* than in *C. glabrata* [31]. Several genes show encoding of ABC transporters and the important families of *C. auris* major facilitator superfamily (MFS) genes [76]. An Indian study with a large number of isolates showed that 41% of *C. auris* from India showed resistance to two antifungal classes and 4% to three antifungal agents [28]. Molecular mechanisms responsible for antifungal resistance point to efflux pumps and mutations in the lanosterol 14-alpha-demethylase (*ERG11*) gene to explain the high rate of resistance to fluconazole [28,31]. The latter study demonstrated that 90% of *C. auris* isolates were resistant to fluconazole (MICs 32 to ≥64 mg/L). *ERG11* sequences of resistant *C. auris* exhibited substitutions of the Y132 and K143 amino acids in 77% of the fluconazole-resistant strains. No substitutions at these positions were observed in isolates with low fluconazole MICs (1–2 mg/L), suggesting that these substitutions confer the fluconazole resistance phenotype similar to that described for *C. albicans* [77]. 

Another study, in a murine model, showed that micafungin was superior compared to fluconazole or amphotericin B, with greater fungicidal activity [78]. These findings make echinocandins the drugs of choice to treat *C. auris* infections and clinical trials are on their way to explore the therapeutic potential of new drugs.

The combination of antifungals such as voriconazole and echinocandins has been shown to be promising in vitro against resistant *C. auris* [79]. Although some studies show variable susceptibility of *C. auris* to the echinocandin class [80], the good news is that there are new drugs in development with excellent activity against *C. auris* [71,72,73,81,82,83,84,85]. SCY-078, a novel orally bioavailable 1,3-β-d-glucan synthesis inhibitor, has been shown to exhibit both in vitro and in vivo activity against *C. auris*, including some echinocandin-resistant isolates. VT-1598 is another new azole drug with broad activity including *C. auris* isolates (MIC range 0.03–8 mg/L) [86,87].

The cleaning and terminal disinfection of rooms where *C. auris*-colonized patients have been problematic [61,88]. Moore et al. [89] showed that chlorine-based disinfectants and iodine-based skin antiseptics were effective against *C. auris* and reduced environmental contamination and skin colonization. Chlorhexidine-based products may also be effective. Abdolrasouli and collaborators [90] demonstrated that *C. auris* isolates were inhibited by chlorhexidine gluconate at 0.125–1.5% and by iodinated povidone at a concentration of 0.07–1.25%.

## 2. *Aspergillus fumigatus*

### 2.1. Azole Resistance in A. fumigatus

*Aspergillus* remains significant as one of the causative agents of invasive infections in immunocompromised individuals and frequently constitutes the most common mold genus isolated in this setting. While voriconazole is the primary drug of choice in the treatment of invasive aspergillosis, the emergence of azole resistance in *Aspergillus* has been a concern since the first report of secondary resistance of *A. fumigatus* to itraconazole in 1997 [91,92]. Antifungal drugs which exert activity against *Aspergillus* spp. are amphotericin B, triazoles, and echinocandins. Furthermore, triazoles are of particular significance due to the availability of oral formulations. Based on this, triazoles constitute significant therapeutic options for patients with chronic pulmonary aspergillosis and allergic bronchopulmonary aspergillosis who require long-term therapy [93] and azole resistance in *A. fumigatus* is thus a concern in this respect as well.

Secondary azole resistance in *A. fumigatus* has been reported from many countries and centers in six continents at extensively varying rates. Similar to those for strains isolated from clinical samples, resistance rates detected for environmental strains are also diverse [94,95,96,97,98,99,100,101]. The ISHAM/ECMM *Aspergillus* Resistance Surveillance Working Group aims to facilitate surveillance studies to determine resistance epidemiology in countries where data are currently lacking and provide further insight in terms of clinical implications [102]. 

While secondary azole resistance in *A. fumigatus* draws remarkable attention, the awareness and knowledge on primary antifungal resistance in *Aspergillus* strains are also increasing. Among the species which are relatively common causes of invasive infections and exhibit primary resistance or reduced susceptibility to one or more antifungal drugs are *Aspergillus lentulus* (resistance to azoles and amphotericin B and varied susceptibility to caspofungin), *Aspergillus flavus* (reduced susceptibility to amphotericin B and varied susceptibility to caspofungin), *Aspergillus alliaceus* (reduced susceptibility to amphotericin B and caspofungin), and *Aspergillus terreus* (resistance to amphotericin B) [96]. 

### 2.2. Detection of Antifungal Resistance In Vitro by Reference Methods

Reference CLSI [103] and EUCAST [104,105] microdilution susceptibility testing methods are available for testing antifungal drugs against *Aspergillus* and recommended for routine use [106,107]. A disk diffusion method of CLSI for testing non-dermatophyte molds and thus applying also to *Aspergillus* is also available [108]. Epidemiological cut-off values have been determined for the interpretation of the results obtained by the CLSI method [109,110,111,112], while both epidemiological cut-off values and clinical breakpoints are available for interpreting EUCAST minimum inhibitory concentration values (MIC, mg/L) for some drugs and species [113]. The official reading method for amphotericin B and azole MICs against *Aspergillus* is visual reading for both CLSI and EUCAST methodologies. A spectrophotometric reading alternative for EUCAST amphotericin B and azole MICs at 5% growth cut-off (vs. complete inhibition of growth visually) proved to be a reliable alternative [105]. 

An agar screening method for the detection of secondary azole resistance in *A. fumigatus* strains has also been validated recently by a multicenter study undertaken by EUCAST [114]. This method uses (in-house or commercially available) 4-well agar plates containing itraconazole (4 mg/L), voriconazole (2 mg/L), and posaconazole (0.5 mg/L); the fourth well serves as the growth control well without any antifungal drug. The ranges of 80–100% and 97–100% were obtained, respectively, for interobserver agreement rate and overall sensitivity. The inter-plate (in-house vs. commercial) agreement rate was high. Similarly, the sensitivity for simulated mixed samples of wild-type and mutant strains and the overall specificity rates also proved to be acceptably high (83–100% and 95–100%, respectively). Based on these data, the assay was validated and is now available as a reference method as documented in EUCAST E.DEF 10.1 [115]. It is an easy and reliable method recommended to be used for routine laboratory work-up, to be followed by reference MIC testing for confirmation in case of the detection of a resistant strain [106].

### 2.3. Molecular Mechanisms Involved in Secondary Azole Resistance and the Resulting Azole Susceptibility Profiles

Point mutations in the *cyp51A* gene associated with amino acid changes of M220, G54, G138, G448S, as well as L98H are the most common mechanisms of secondary azole resistance in *A. fumigatus*. Extra copies of the *cyp51A* gene (e.g., tandem repeats of a 34- or 46-bp sequence in the promoter of the *cyp51A* gene) may also accompany specific amino acid changes. The typical examples of this combined pattern are TR34/L98H and TR46/Y121F/T289A [116]. A tandem repeat of 53 bp without any accompanying amino acid change has also been described [117,118]. In addition, non-*cyp51* mutations and increased expression of efflux pumps may play a role in the development of secondary azole resistance. On the other hand, the mechanism remains unknown for a number of isolates. The expected azole susceptibility profiles in relation to the associated amino acid changes and/or tandem repeats are summarized in Table 1 [95,116]. 

### 2.4. Acquisition of Secondary Azole Resistance

There are two mechanisms that play a role in the development of secondary azole resistance in *A. fumigatus*. First is the (long-term) azole therapy in an individual patient with chronic pulmonary lung disease mostly in existence of a pulmonary cavity, and second is the direct acquisition of a resistant strain from the environment. The latter develops due to the use of azole fungicides (penconazole, difenoconazole, tetraconazole, and tebuconazole) in the environment in agriculture for plant protection [98,121]. The molecular mechanisms leading to resistance also differ in general for these two routes of acquisition. In the patient-acquired route, M220, G54, and G138 changes are more common while TR34/L98H and TR46/Y121F/T289A patterns are mostly (but not always) observed following environmental acquisition [95,116]. 

### 2.5. Clinical Implications and Current Recommendations for Treatment of Aspergillosis due to Azole-Resistant A. fumigatus

While high azole MICs [122,123] or the existence of *cyp51A* mutations [124] were found to be correlated with clinical failure in some studies, other investigators were not able to detect any correlation between MICs and survival rates [125]. This may also emphasize the influence of host factors as well as several others on clinical outcomes in invasive fungal infections observed in immunocompromised individuals. The low rates of resistance, i.e., the low number of infections due to azole-resistant strains included in the analysis, may also render it more difficult to detect any possibly existing in vitro–in vivo correlation. “Strong” or “Moderate” recommendations for the treatment of documented azole-resistant aspergillosis, as included in the recently published ESCMID-ECMM-ERS Guideline [106], are liposomal amphotericin B monotherapy (Strength of Recommendation (SoR) and Quality of Evidence (QoE): AIIu) and voriconazole and anidulafungin combination (BIII), respectively. Other options with a “Marginal” level of recommendation (CIII for all noted alternatives) include amphotericin B lipid complex monotherapy, posaconazole and caspofungin combination, caspofungin or micafungin monotherapy. The expert opinion, on the other hand, recommends a modification in primary therapeutic choice of voriconazole in case of local environmental resistance rates of >10%. Voriconazole and echinocandin combination or liposomal amphotericin B monotherapy is recommended for initial therapy under these settings [126]. 

## 3. Concluding Remark

The emerging field of molecular mechanisms of antifungal resistance has been an underestimated area of global public health concern, but significant progress has been made lately in *A. fumigatus* and *C. auris*, although research challenges remain formidable.

## Figures and Tables

**Table 1:** Expected azole susceptibility profiles with respect to the detected resistance mechanism(s).

Associated Amino Acid Change/Tandem Repeat	Resistance	Reduced Susceptibility	Variable Susceptibility Profile
G54	ITC, POS		
G138	ITC, POS		
G448S	VRC	ITC, POS	
M220	ITC	VRC	POS
TR34/L98H *	ITC, VRC, POS, ISV		
TR46/Y121F/T289A	VRC		ITC, POS
TR53	ITC, VRC	POS	

ISV: isavuconazole; ITC: itraconazole; POS: posaconazole; VRC: voriconazole; *: Isolates with TR34/L98H/S297T/F495I changes may have lower minimum inhibitory concentrations (MICs) of voriconazole in the wild-type range. The S297T mutation might be a compensatory mutation in these cases [119,120].
